# Green Process: Improved Semi-Continuous Fermentation of *Pichia pastoris* Based on the Principle of Vitality Cell Separation

**DOI:** 10.3389/fbioe.2021.777774

**Published:** 2021-11-30

**Authors:** Denggang Wang, Wenjie Li, Xinying Zhang, Shuli Liang, Ying Lin

**Affiliations:** South China University of Technology, Guangzhou, China

**Keywords:** semi-continuous cultivation, pichia pastoris, sedimentation, vitality cells, metal ion

## Abstract

The large-scale fermentation of *Pichia pastoris* for recombinant protein production would be time consuming and produce a large amount of waste yeast. Here we introduce a novel semi-continuous fermentation process for P. pastoris GS115 that can separate vitality cells from broth and recycle the cells to produce high-secretory recombinant pectate lyase. It is based on differences in cell sedimentation coefficients with the formation of salt bridges between metal ions and various cell states. Compared to batch-fed cultivation and general semi-continuous culture, the novel process has significant advantages, such as consuming fewer resources, taking less time, and producing less waste yeast. Sedimentation with the addition of Fe3+ metal ions consumed 14.8 ± 0.0% glycerol, 97.8 ± 1.3% methanol, 55.0 ± 0.9 inorganic salts, 81.5 ± 0.0% time cost, and 77.0 ± 0.1% waste yeast versus batch-fed cultivation to produce an equal amount of protein; in addition, the cost of solid–liquid separation was lower for cells in the collected fermentation broth. The process is economically and environmentally efficient for producing recombinant proteins.

## Introduction

*Pichia pastoris* has been used to yield various recombinant proteins. According to the web platform www.pichia.com, more than 5,000 different proteins have been produced using this system (Schwarzhans et al., 2017). As a microorganism that is generally considered to be safe, it has been applied to produce pharmaceutical proteins due to its high yield of recombinant proteins with high similarity in terms of glycosylation to mammalian cells (Vinayagamurthy et al., 2007; Ahmad et al., 2014). Other great advantages of *P*. *pastoris* are the production of high levels of heterologous proteins and the fact that it grows in minimal medium at high density with low levels of endogenous protein secretion (Cereghino and Cregg, 2000; Zhan et al., 2014). Its use in high-cell-density batch-fed fermentation in automatically controlled bioreactors has attracted attention in recent years (Liu et al., 2020a; Liu et al., 2020b; Zhang et al., 2020; Zhou et al., 2020). According to “Pichia Fermentation Process Guidelines,” high-cell-density fermentation (HCDF) can be divided into three phases: the glycerol batch phase, glycerol fed-batch phase, and methanol induction phase (Sun et al., 2018; Viña-Gonzalez et al., 2018). The first two constitute the growth stage that uses glycerol as a C-source to obtain high biomass; the wet cell weight (WCW) can be as much as 300 g/l or higher (Liu and Zhu, 2015; Dagar and Khasa, 2018). The general methanol induction phase can be further divided into methanol adaptive and methanol induction phases. Because accumulated methanol is cytotoxic for *P*. *pastoris* (Juturu and Wu, 2018; Liu et al., 2019), the adaptive phase of the fermentation process should be set to ensure that enough alcohol oxidase is produced to provide methanol metabolic capacity and prevent it from accumulating during prophase induction. The growth stage is nonproductive and generally accounts for about 30% of the whole cycle (Minning et al., 2001; Jahic et al., 2003). Furthermore, the methanol adaptive phase is an inefficient production phase, which will cost 8–10 h (about 5%–10% of the whole production cycle). If the non-production and inefficient production stages could be shortened or removed, productivity would significantly improve. Moreover, a greener and sustainable process would reduce the consumption of water, energy, and associated raw material. It is common to replace original fed-batch culture with continuous (Rahimi et al., 2019a; Rahimi et al., 2019b; Nieto-Taype et al., 2020) or semi-continuous (Pfeffer et al., 2006; Borchert et al., 2015) culture.

Continuous cultivation has more often been applied to the basic physiology of wild-type cells (Peebo and Neubauer, 2018). Although it is also used to manufacture recombinant proteins (Marschall et al., 2016; Kateja et al., 2017), applying chemostat cultivation to each new process can be laborious and time-consuming (Daran-Lapujade et al., 2009; Peebo and Neubauer, 2018). Another problem is that continuous cultivation must be maintained in a steady state for a long time, which means that any change, for example, contaminative microbes, changes in dissolved oxygen (DO) due to fluctuations in instrumentation, and volume changes due to evaporation or acid–base regulation, can lead in an undesirable direction. That is to say, continuous cultivation for the manufacture of recombinant proteins requires increased investment in equipment and high skill levels from expert technical personnel.

Semi-continuous cultivation (SCC) entails periodic removal of the cultivation fraction and replacement with an equal volume of fresh cultivation medium; each dilution returns the cultivation to approximately the same cell density after the previous one (Henley, 2019). This process dramatically dilutes the cell population, reduces the number of producers, and requires additional resources to be devoted to the growth of cells. Many researchers have been working to solve these problems to achieve cell recycling, through processes such as centrifugal separation (Gledhill et al., 1973), membrane separation (von Weymarn et al., 2002; Krusong and Vichitraka, 2011), and immobilization technology (Chia et al., 2008; Jiang et al., 2009). However, all of these methods have the same disadvantage: it is difficult to divide active and inactive cells, which gradually increases the fraction of inactive cells and decreases productivity with an increase in the number of cycles (Gledhill et al., 1973; Chia et al., 2008).

The high density of cells produced by *P*. *pastoris* is an important advantage in the production process; it can reach up to 150 g cell dry weight (DCW) per liter of fermentation broth in batch-fed cultivation in a bioreactor (Jahic et al., 2006), with a solid–liquid ratio close to 1:1 (Looser et al., 2015). However, the mass cells also increase the load of liquid–solid separation, resulting in a large number of discarded cells, recognized as semi-solid waste (Cheng et al., 2020). This imposes a massive burden on the environment. Metal ions can accelerate flocculation in yeast cells. Meanwhile, Fe^3+^ and Ca^2+^ are two ideal flocculants (Soares and Seynaeve, 2000; Gao et al., 2016). Because flocculation ability differs in different yeast cells, adding Fe^3+^ and Ca^2+^ to promote *P*. *pastoris* flocculation can accelerate the sedimentation of cells and divide the cells into active and inactive fractions.

In this work, three kinds of semi-continuous cultivation were performed, including general semi-continuous cultivation (SCC), natural sedimentation (about 24 h) to recycle cells for semi-continuous cultivation (NS24), and semi-continuous cultivation with metal ions (Ca^2+^ or Fe^3+^; CCa and CFe, respectively). We successfully applied these methods to produce proteins with active *P*. *pastoris* cells from broth and achieved stable, optimal operation for more than month.

## Experimental

### Strains and media

The *P*. *pastoris* GS115 strain containing the pPICHKAPel vector was used for the heterologous expression of pectate lyase (Pel from Bacillus sp. RN1) under control of the AOX1 promoter G/Pel (Zheng et al., 2020). The *P*. *pastoris* GS115 strain containing the pPICZαAphy vector was used for heterologous expression of phytase (Phy from C. amalonaticus CGMCC 1696) under the control of the AOX1 promoter G/Phy (Weidner et al., 2010). The pre-inoculum for bioreactor cultivation was yeast extract peptone dextrose.

### Semi-continuous cultivation

The SCC experiment was conducted in a 3-l Sartorius Biostat A Plus device (Sartorius Biotech, Gottingen, Germany). Initial and replacement media consisted of basal salt medium (BSM) added at 4.35 ml trace element solution PTM_1_ per liter as described elsewhere (Zhao et al., 2008), and the inoculation concentration was 8% (v/v). BSM contained 27 ml·l^−1^ 85% H_3_PO_4_, 40 g·l^−1^ glycerol, 18 g·l^−1^ K_2_SO_4_, 14.9 g·l^−1^ MgSO_4_·7 H_2_O, 4.13 g·l^−1^ KOH, and 0.93 g·l^−1^ CaSO_4_, supplemented with 4.35 ml·l^−1^ PTM1 trace salts containing 65 g·l^−1^ FeSO_4_·7H_2_O, 20 g·l^−1^ ZnCl_2_, 6 g·l^−1^ CuSO_4_·5H_2_O, 3 g·l^−1^ MnSO_4_·H_2_O, 0.5 g·l^−1^ CoCl_2_, 0.2 g·l^−1^ MoNa_2_O_4_·2H_2_O, 0.2 g·l^−1^ biotin, 0.09 g·l^−1^ KI, 0.02 g·l^−1^ H_3_BO_3_, and 5.0 ml·l^−1^ 98% H_2_SO_4_.

**FIGURE 1:**
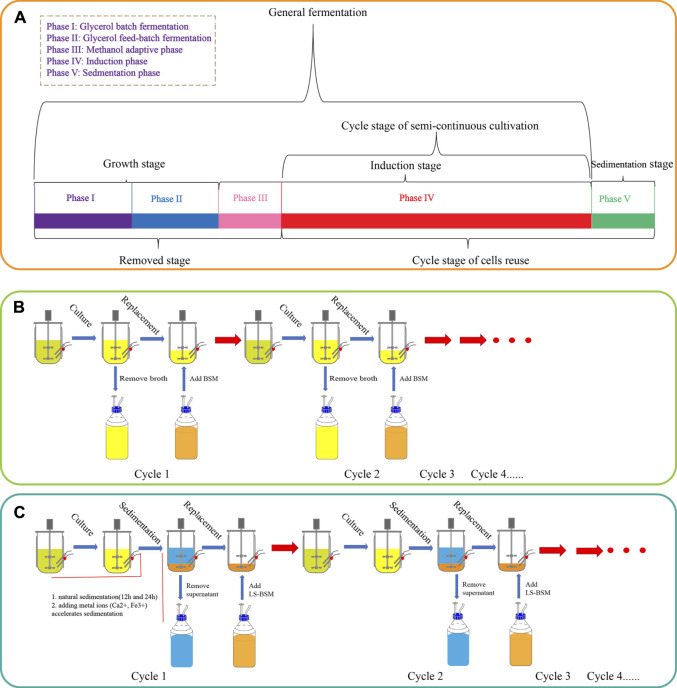
The process flow of *P*. *pastoris*. **(A)** Division of *P*. *pastoris* culture stages. General batch-fed cultivation is divided into four stages. Semi-continuous cultivation repeats phase IV (induction stage). The new process that reuses cells adds a sedimentation stage (phase V) to help cycle cells, and phase IV and phase V are cyclic. **(B)** A schematic diagram of semi-continuous cultivation. **(C)** A schematic diagram of the new process, including natural sedimentation and the addition of metal ions (Ca^2+^ and Fe^3+^) to accelerate sedimentation.

The fermentation process was divided into four phases, as shown in Figure 1. Phases I and II were the cell growth stage in which DO was maintained at >20% by controlling the agitation rate (200–1,000 rpm, phase I), with a feed rate of 50% (w/w) glycerol containing 12 ml·l^−1^ PTM1 solution (phase II); temperature was maintained at 30°C. pH was maintained at 5.5 by feeding with 25% ammonium hydroxide. Phases III and IV were the induction stages, in which methanol containing 12 ml·l^−1^ PTM1 solution was fed to produce target proteins. The temperature was controlled at 25°C, pH was adjusted to 6.0, and DO is maintained at >20%. All SCCs followed the same procedure in the first cycle as general batch-fed cultivation. However, at the end of the first cycle, part of broth was removed so that 0.75 l remained in the bioreactor, and then BSM was added until reaching a total volume of 1.5 l. After the second cycle, the above operation was repeated. This process removed phases I, II, and III, leaving only phase IV. The SCC of reused cells has an additional sedimentation stage (phase V) for distinguishing and collecting cells. At the end of phase V, the supernatant was removed, leaving only 0.35 l fermentation broth (15% of the total fermentation broth). Then, low-salt basal salt medium (LSM) containing 4.35 ml PTM1 per liter was added to a total volume of 1.5 l. The LSM contained 27 ml·l^−1^ 85% H_3_PO_4_, 9 g·l^−1^ K_2_SO_4_, 7.45 g·l^−1^ MgSO_4_·7 H_2_O, 2.065 g·l^−1^ KOH, and 0.465 g·l^−1^ CaSO_4_.

### Biomass analysis

Sample (10 ml) were collected every 4 h (phases I and II) or 6 h (stage III) and characterized by absorbance at 600 nm in 2 ml cuvettes.

### Measurement of pectate lyase

Pectate lyase activity was determined according to the method described by Zheng et al. (2020), with one modification: measuring the absorbance change at 235 nm with 2 mg PGA ml-1 as the substrate in 50 mM glycine–NaOH (pH 10.0) buffer containing 1 mM CaCl_2_ for 40°C, 30 min. One unit (U) of pectin lyase activity was defined as the amount of enzyme that is required to produce unsaturated oligogalacturonide equivalent to 1 μmol of unsaturated diglucuronide min–1 using a molecular extinction coefficient of 4,600 M^−1^ cm^−1^ at 235 nm (Zheng et al., 2020).

### Measurement of propidium iodide staining

A 1-ml cell resuspension (OD_600_ = 40) was centrifuged at 6,000 rpm for 1 min. The cells were collected and washed three times in 10 mM phosphate-buffered saline (PBS, pH 7.4), and OD_600_ was adjusted to 5. Then, a 10-μl aliquot of 5 mmol propidium iodide (PI) was added to 200 µl cell suspension and incubated on a shaker for 20 min at 37°C. The cells were washed three times and resuspended in 1.5 ml PBS (pH 7.4). The cell suspension was analyzed by flow cytometry (Beckman Coulter, Fullerton, CA, USA). One hundred thousand cells per sample were counted and analyzed using Exp032 software (Beckman Coulter). The mean value of fluorescence intensity (X-mean) of the sample could be calculated by software FlowJo v10.

## Results and discussion

### Semi-continuous cultivation

**FIGURE 2:**
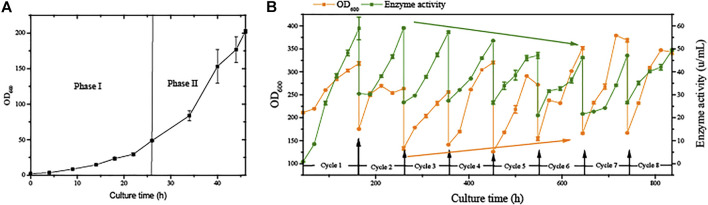
Growth stage and semi-continuous cultivation of G/Pel. **(A)** Growth stages, including phase I and phase II. **(B)** Growth curve and enzyme activity curve of G/Pel cells during semi-continuous cultivation.

We developed an improved, greener, and more energy-efficient cultivation process for recombinant protein production from Pichia pastoris. In regular semi-continuous cultivation (SSC), the period of fermentation was shortened by omitting the growth phase (Figure 2) after the second cycle. This time would be further prolonged if higher cell concentrations are used for initial induction, combined with the methanol adaptive phase (phase III).

**FIGURE 3:**
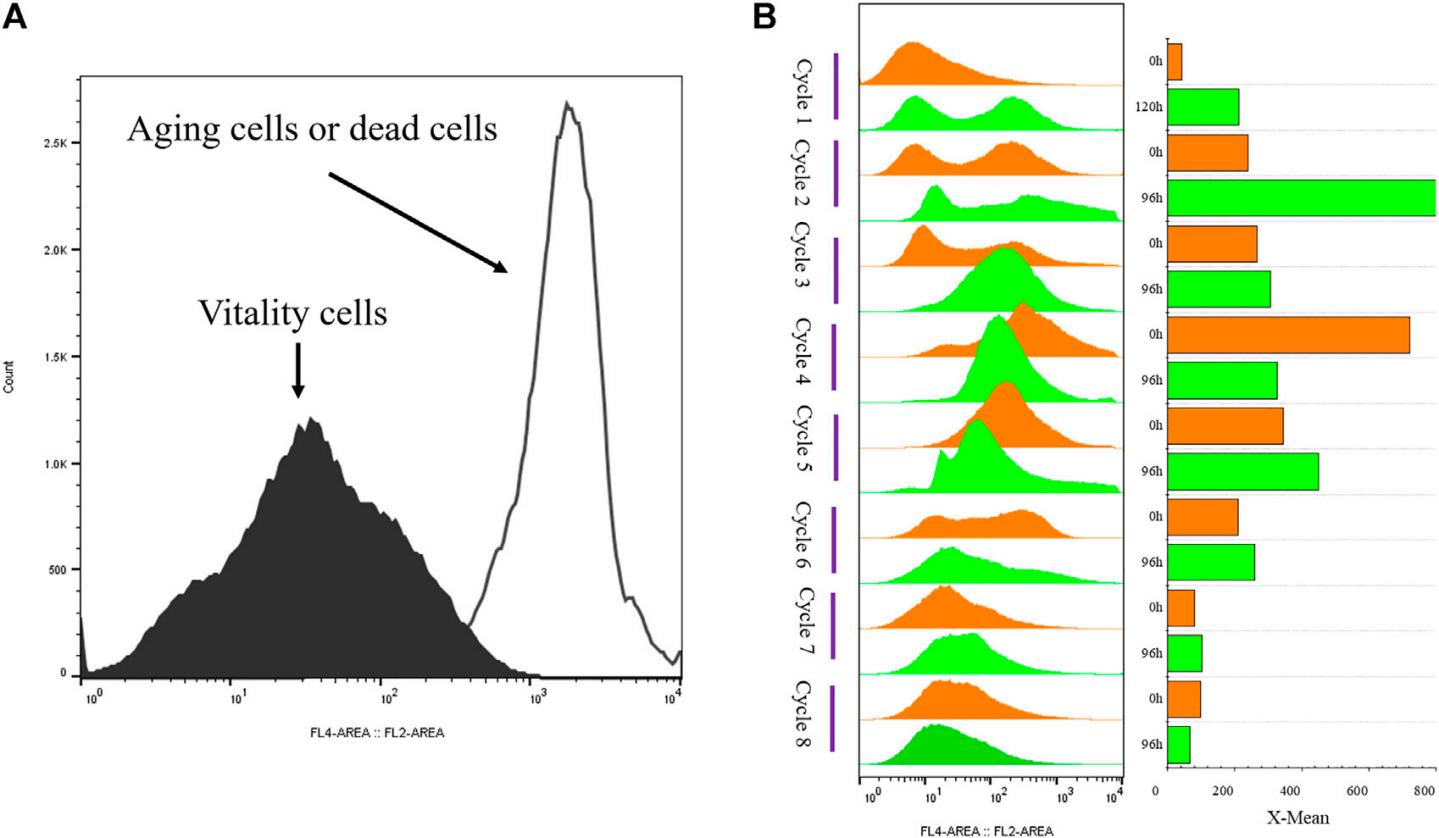
Histogram of flow cytometry and cell vitality of G/Pel. **(A)** Negative and positive histogram of flow cytometry. **(B)** G/Pel cell vitality after semi-continuous cultivation. The left side shows a histogram of flow cytometry, and the histogram migrates to the right, with increased fluorescence and decreased cell vitality. The right side shows the statistical data of fluorescence detected by flow cytometry at different time points. Y-axis: fermentation cycles; each cycle contains two points. 0 h (orange), the viability of remaining cells; 120 or 96 h (green), the cell viability at the end of the fermentation cycle. X-axis: the cell viability values.

The strain G/Pel was used to produce pectate lyase in a 3-l bioreactor after the growth stage, which was evaluated by running eight cycles. The levels of pectate lyase gradually decreased within the eight cycles. As the cycle changed, the production capacity gradually declined. At the end of the fifth cycle, pectinase activity was only 79% of the first cycle. The patterns of cell growth were the opposite of those of enzyme activity. From the second cycle, cell growth gradually accelerated. Although the number of cells gradually increased, the number of vitality cells gradually declined (Figure 3), accounting for the gradual decline in production capacity. Similar results were observed for phytase production (Supplementary Figure S1), and the production capacity decreased even more. which was due to reduced productivity as cells age (Verbelen et al., 2009). At the end of the fourth cycle, the enzyme activity was only 23.8% of that in the first cycle.

However, the mass of cells was drained from the broth each cycle, and the vitality cells were difficult to separate from the remaining broth, leading to the accumulation of aging cells. Dead cells would inevitably affect productivity.

### Semi-continuous cultivation with natural sedimentation to recycle cells

**FIGURE 4:**
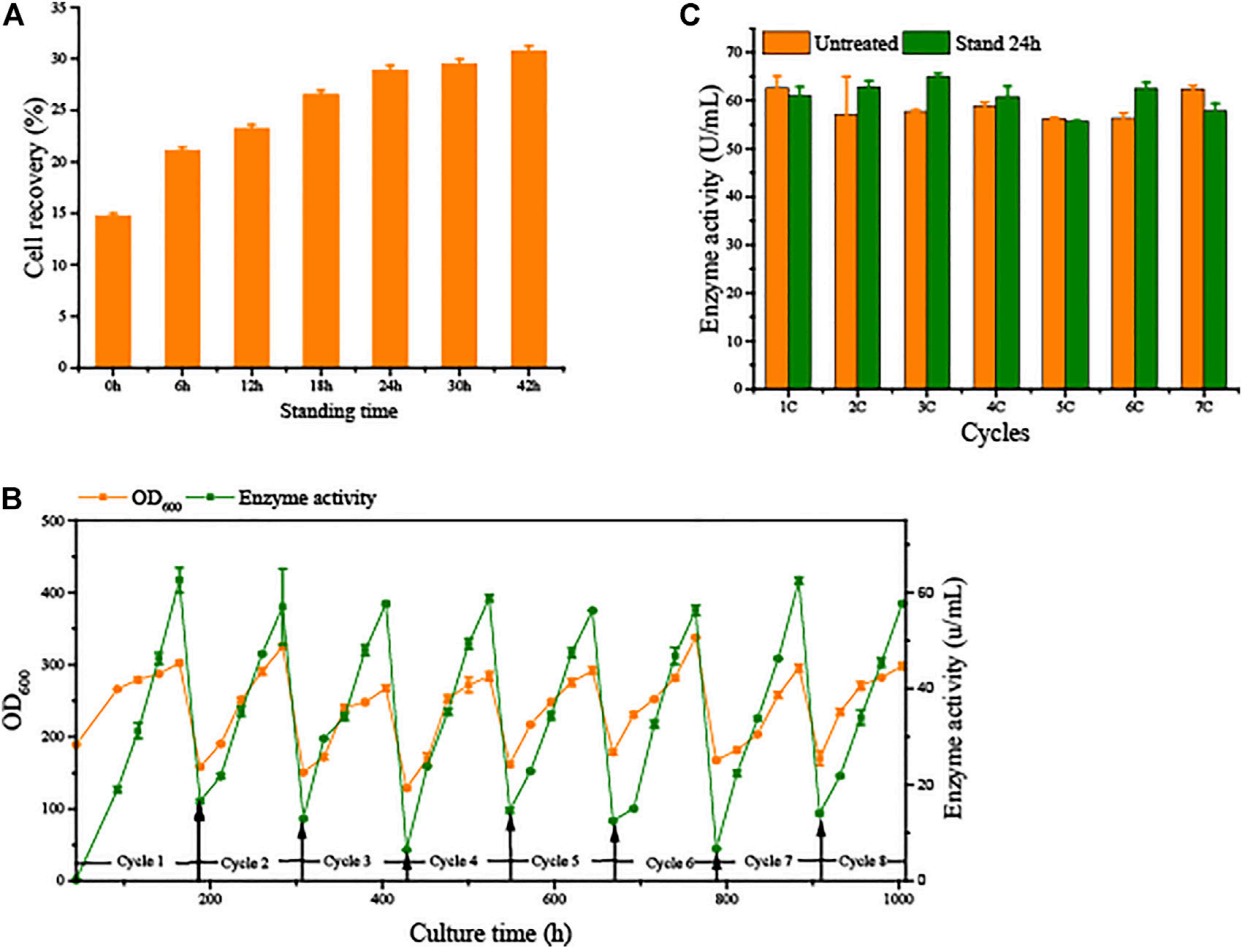
Natural sedimentation to recycle cells for semi-continuous cultivation. **(A)** Cell recovery of G/Pel at different sedimentation times. **(B)** Growth curve and enzyme activity curve of semi-continuous cultivation of G/Pel for natural sedimentation for 24 h to recycle cells. **(C)** Comparison between the untreated group and after sedimentation to recycle cells.

To avoid the significant decrease in production capacity due to cell aging in SCC, we conducted semi-continuous cultivation with sedimentation to separate vitality cells from the broth. Sedimentation time is a principal element. As shown in Figure 4, the longer the sedimentation time, the better the separation of cells. Remaining in the lowest layer by 15% of fermentation broth to collect cells, the cell recovery reached 28.9 ± 0.45% after 24 h of sedimentation. Over time, the cell recovery rate slowly increases. These results indicate that the G/Pel cells were better for precipitation of solid/liquid separation when the fermentation broth stood for 24 h.

**FIGURE 5:**
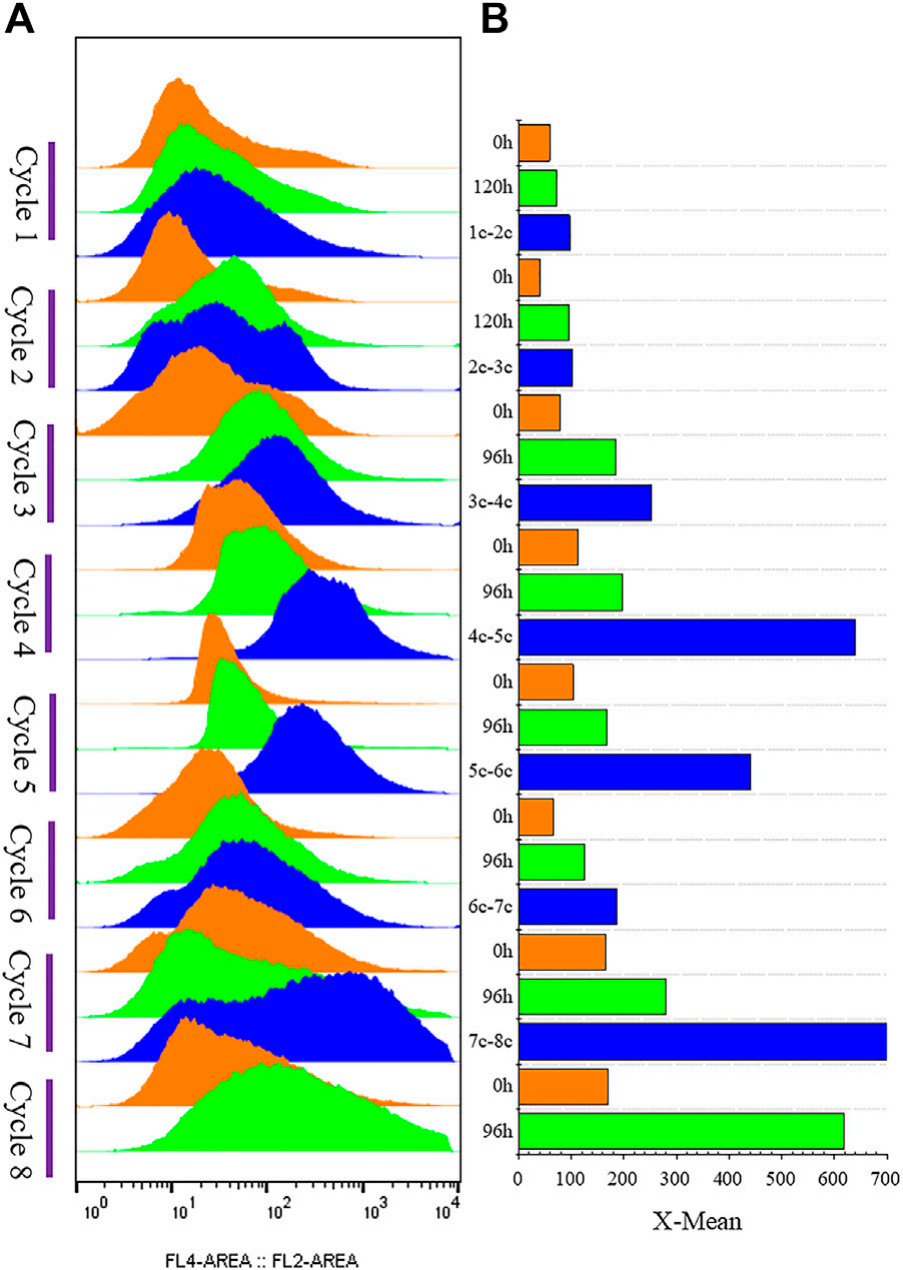
G/Pel cell vitality after natural sedimentation for 24 h to recycle cells for semi-continuous cultivation. **(A)** Shows a histogram of flow cytometry, and the histogram migrates to the right, with increased fluorescence and decreased cell vitality. **(B)** Shows the statistical data of fluorescence detected by flow cytometry at different time points. **Y-axis:** fermentation cycles; each cycle contains three points. 0 h (orange), the viability of remaining cells (precipitated cells); nc-n+1c (blue), the viability of cells was removed from the previous cycle; 120 or 96 h (green), the cell viability at the end of fermentation cycle. X-axis: the cell viability values.

Moreover, 15% fermentation broth remained in the bottom to collect cells, which were then added to fresh LSM for the subsequent fermentation cycle. The average cell recovery reached 39.5 ± 4.4%. The mass of G/Pel cells grew to reach about 300 OD_600_, and the maximum production of pectate lyase was about 60 U/ml until the eighth cycle (Figure 4). The growth of cells and pectate lyase production were stable in each cycle because aging cells were discarded (Figure 5). Moreover, most vitality cells remained for the next cycle. There was almost no loss of pectate lyase activity after standing for 24 h, indicating that the process can be applied to culture G/Pel (Figure 4). In addition, the same process was used for the production of phytase, with almost identical results (Supplementary Figure S2). Compared to fed-batch fermentation, we saved 140 h and substantial water and electricity within the eight cycles. However, fermentative broths need to stand for 24 h between cycles, which is time consuming and increases the risk of contamination. The results were similar to Pfeffer et al. (2006); the semi-continuous process would collect more products and reduce.

### Semi-continuous cultivation with metal ion sedimentation to recycle cells

**FIGURE 6:**
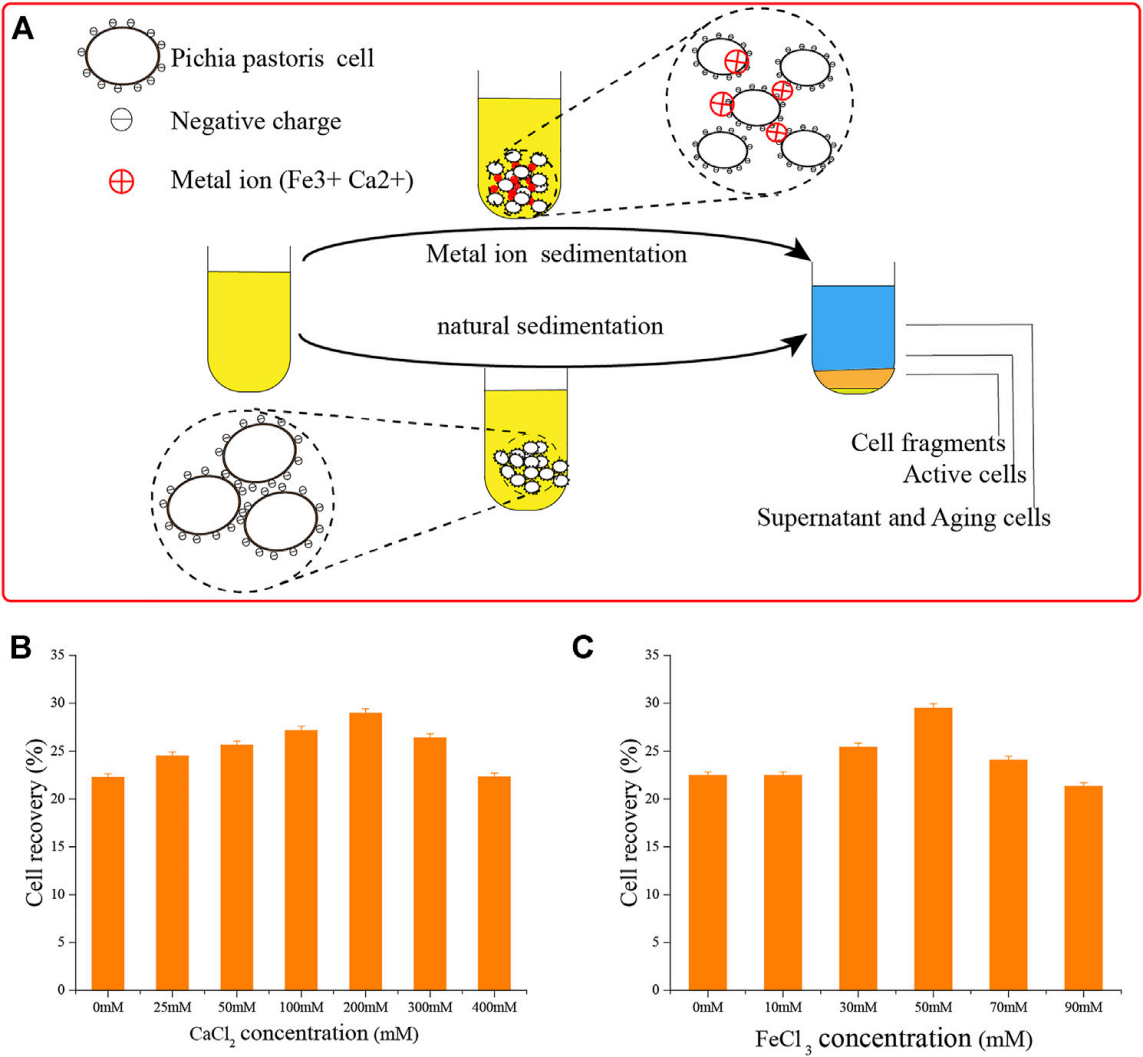
Adding metal ions to accelerate cell sedimentation. **(A)** Schematic diagram of adding metal ions to accelerate cell sedimentation and natural cell sedimentation. **(B)** Adding Ca^2+^ ions to accelerate cell sedimentation. **(C)** Adding Fe^3+^ ions to accelerate cell sedimentation.

Yeast cells have a negative surface charge (Figure 6A) (White and Walker, 2011; Kregiel et al., 2012), which affect the sedimentation efficiency of yeast due to the repulsion of the same charge (Benabdesselam, 2010; Tibayrenc et al., 2011). The positive charges of metal ions can interact with the cell surface to form a salt bridge (Mill, 1964; Jayatissa and Rose, 1976; O'Mahony et al., 2006; Goossens et al., 2015). We applied this concept, using Ca^2+^ and Fe^3+^ ions.

To deliver these ions, we added CaCl_2_ and FeCl_3_. First, we determined their optimal concentrations. As shown in Figure 6, the effects of flocculation precipitation of *P*. *pastoris* cells increased over 12 h with the concentration of Ca^2+^ ions. When the concentration of CaCl_2_ was 200 mM, the supernatant was clarified and sedimentation was the best. Cell recovery reached 29.0 ± 0.45%, almost as good as natural sedimentation for 24 h. Using the same process, we found that 50 mM FeCl_3_ was optimal.

**FIGURE 7:**
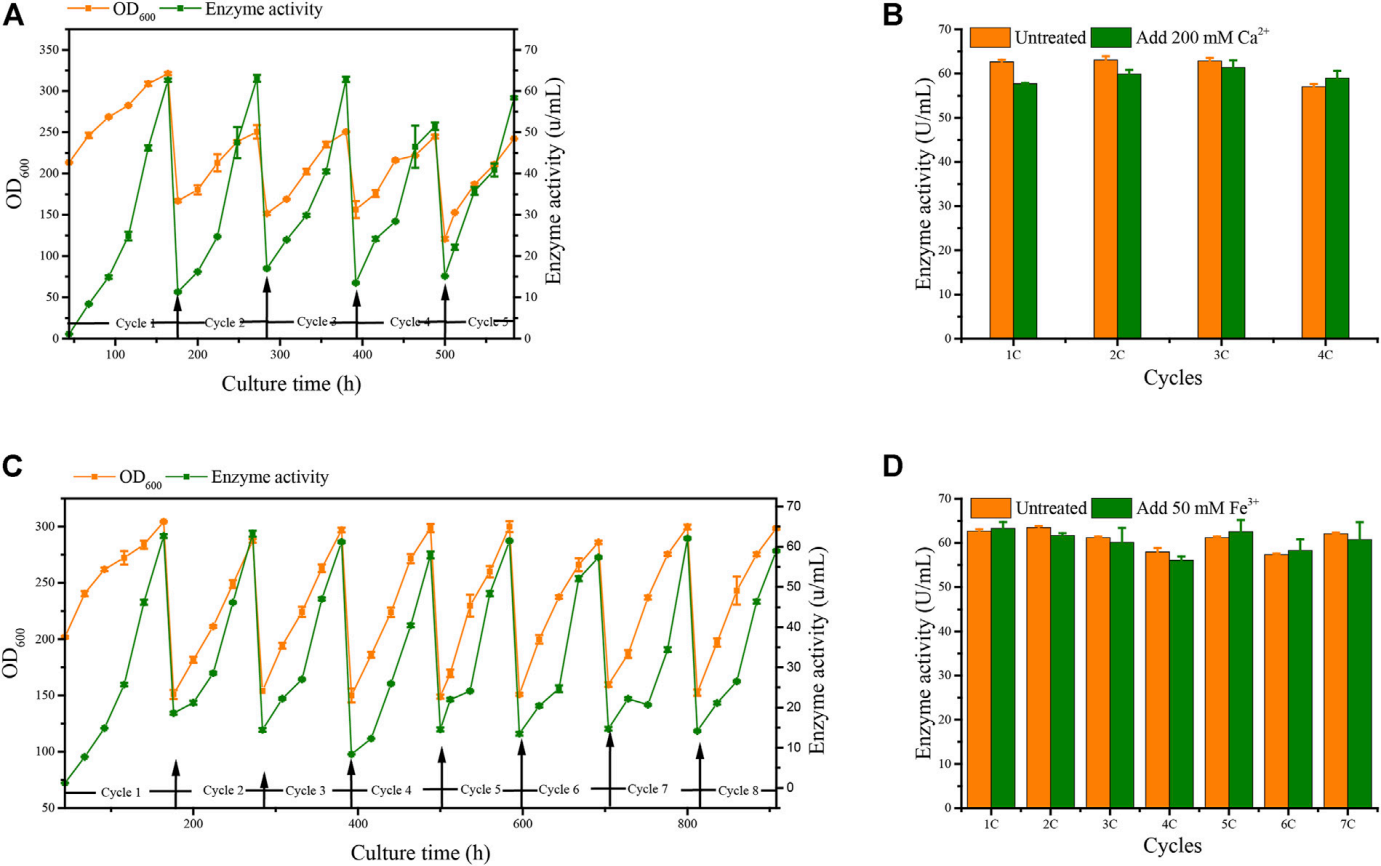
Adding metal ions to accelerate cell sedimentation to recycle cells for semi-continuous cultivation. **(A)** Growth curve and enzyme activity after adding 200 mM Ca^2+^ ions to accelerate G/Pel sedimentation to recycle cells. **(B)** Comparison between the untreated group and after adding 200 mM Ca^2+^ ions to accelerate G/Pel sedimentation to recycle cells. **(C)** Growth curve and enzyme activity curve after adding 50 mM Fe^3+^ ions to accelerate G/Pel sedimentation to recycle cells. **(D)** Comparison between the untreated group and after adding 50 mM Fe^3+^ ions to accelerate G/Pel sedimentation to recycle cells.

**FIGURE 8:**
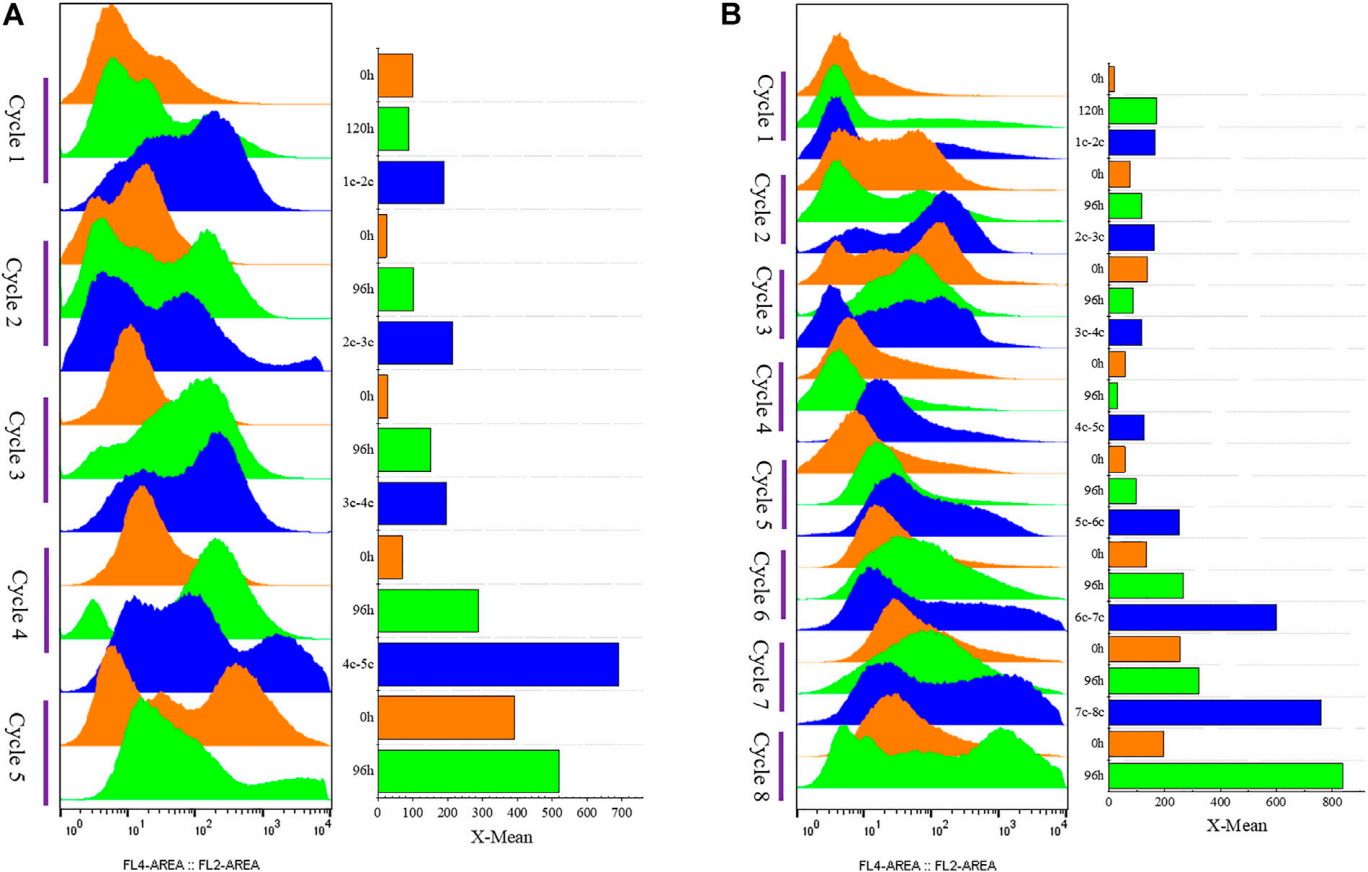
Cell vitality of G/Pel. Y-axis: fermentation cycles; each cycle contains three points. 0 h (orange), the viability of remaining cells (precipitated cell); nc-n+1c (blue), the viability of cells was removed from the previous cycle; 120 or 96 h (green), the cell viability at the end of the fermentation cycle. X-axis: the cell viability values. **(A)** Semi-continuous cultivation with 200 mM Ca^2+^ ions to accelerate G/Pel sedimentation to recycle cells. **(B)** Semi-continuous cultivation with 50 mM Fe^3+^ ions to accelerate G/Pel sedimentation to recycle cells. The left side shows a histogram of flow cytometry, and the histogram migrates to the right, with increased fluorescence and decreased cell vitality. The right side shows the statistical data of fluorescence detected by flow cytometry at different time points.

We applied these to accelerate G/Pel sedimentation for SCC. As shown in Figure 7, the sedimentation rate of G/Pel was significantly accelerated. After sedimentation for 12 h, the layer of vitality cells was collected for the next fermentative cycle, in which the cell density attained about 150 OD_600_ at a total volume of 1.5 l. The average cell recovery was 40.7 ± 6.5% and 38.2 ± 2.5% using Ca^2+^ and Fe^3+^, respectively, almost the same as natural settling for 24 h. However, the effects of Ca^2+^ ion precipitation did not meet expectations. After three cycles, the growth of cells and the production of pectin lyase decreased because Ca^2+^ ions accumulated as the culture was recycled. SCC obtained the best results with Fe^3+^ ion sedimentation. When a fermentative cycle was finished in 96 h, 2 M Fe^3+^ ions were added to the broth, maintained at a concentration of 50 mM ions (2.25 l in the ending fermentation), and then used to accelerate G/Pel cell sedimentation for 12 h. The vitality cells (Figure 8B) were collected for recycle fermentation, and the supernatant was used for pectate lyase preparations. Running eight cycles, the process showed optimal semi-continuous fermentation (Figure 7). It also cut down on processing time and the use of resources, water, and electricity. When SSC with Fe^3+^ ions was applied to the G/Phy strain, similar results were obtained (Supplementary Figure S3).

### Evaluation of efficiency

To demonstrate the benefits of cell recycling, the economic and environmental efficiency of all processes was evaluated from the viewpoint of resource consumption, time cost, production efficiency, and waste cells produced. The product recovery, cell recovery, cell concentration of broth, yield of methanol in cells γx/m, yield of methanol in phytase γ_P/m_, and time cost of each cycle are given in Supplementary Table S1.

The average product recovery of SCC was about 71.5% lower than 88.1%, 87.6%, and 87.2% of NS24, CCa, and CFe. That means that the improved process can collect more protein products in one cycle. The average cell recovery of SCC was about 37.4%, versus 38.8%, 41.2%, and 38.2% for NS24, CCa, and CFe, respectively. In other words, the number of cells collected by sedimentation could meet the production requirements of the next cycle as in standard SCC. The cell concentration of the supernatant was used to evaluate the effects of the process on downstream solid–liquid separation; a lower cell concentration of the supernatant would help reduce the cost of later separation. The results showed that the order of average cell concentration (OD_600_) of the supernatant was CCa (156.1 ± 22.0) < NS24 (161.3 ± 11.8) < CFe < SCC (168.0 ± 13.8) < batch-fed cultivation (311.5 ± 47.1). The concentrations of vitality cells were much lower than those of batch-fed cultivation and the SCC process, indicating that it could effectively reduce the cost of downstream solid–liquid separation.

The biomass on methanol yield coefficient γx/methanol and product on methanol yield coefficient γ_P/methanol_ were used to evaluate the utilization efficiency of methanol. The average γ_x/methanol_ value of SCC, NS24, CCa, and CFe was about 0.178 ± 0.08 g·DCW·g^−1^ methanol^−1^ in the first cycles. In other words, the γ_x/methanol_ of G/Pel in batch-fed cultivation was 0.178 ± 0.08 g·DCW·g^−1^ methanol^−1^. Except for CCa, the γ_x/methanol_ of other processes was larger than the first cycle in the subsequent cycle. Almost all of the values were above 0.22. The average γ_P/methanol_ of SCC, NS24, CCa, and CFe was about 273.5 ± 7.5 u·g^−1^ methanol^−1^ in the first cycles. The γ_P/methanol_ of SCC started to go down after the second cycle, and the processes maintained high γP/methanol values, all greater than or equal to the first cycle, and an average γx/methanol of 281.2 ± 23.4, 284.5 ± 17.4, and 285.5 ± 13.8 u·g^−1^ methanol^−1^, respectively. These results indicate that all processes increased product recovery and the utilization efficiency of methanol.

Next, SCC, NS24, CCa, and CFe were used to conduct a comprehensive evaluation of the entire process of G/Pel culture, as shown in Table 1. The first cycle was batch-fed cultivation, set as a standard to evaluate the different processes. The consumption of glycerol, methanol, inorganic salts, the time cost, and the amount of waste yeast were compared under the condition of producing the same amount of phytase. The consumption rates of methanol glycerol and inorganic salt were 19.7 ± 1.4% of batch-fed cultivation, respectively, and the amount of waste yeast produced and time cost were almost the same as batch-fed cultivation. When producing the same pectate lyase, methanol consumption even reached 130% of batch-fed cultivation. SCC has no apparent efficiency advantages. However, NS24 had a significant advantage over SCC and batch-fed cultivation in terms of vitality cell collection. When producing the same amount of pectate lyase, NS24 only needed 15.2 ± 0.2% glycerol, 56.3 ± 0.1%, 86.3 ± 0.1% time cost, and produced 78.8 ± 0.6% waste yeast. Although methanol consumption was not lower, NS24 was more economical and greener. Adding Ca^2+^ to accelerate G/Pel sedimentation was not suitable for recycling. Although the process reduces glycerol consumption, inorganic salt consumption, time cost, and amount of waste yeast, the decrease in the growth rate of G/Pel meant that the process could not be run for long. CFe further reduced the time cost based versus NS24, which only cost 81.5 ± 0.0% time of batch-fed cultivation while producing the same pectate lyase, less 86.3 ± 0.1% of NS24.

**TABLE 1:** Assessment of G/Pel for three processes.

	Glycerol consumption	Methanol consumption	Inorganic salt consumption	Waste yeast	Time cost
Batch-fed cultivation^a^	100	100	100	100	100
SCC^b^	19.7 ± 1.4	130.1 ± 10.4	88.7 ± 7.0	105.8 ± 7.4	98.6 ± 7.9
NS24^c^	15.2 ± 0.2	100 ± 1.1	56.3 ± 0.1	78.8 ± 0.6	86.3 ± 0.1
CCa^d^	24.0 ± 0.1	101.0 ± 0.1	61.1 ± 0.4	65.7 ± 1.7	85.6 ± 0.5
CFe^e^	14.8 ± 0.0	97.8 ± 1.3	55.0 ± 0.9	77.0 ± 0.1	81.5 ± 0.0

a The consumption and waste yeast emissions, Batch fed-culture as 100% and other process compared to it.

b SCC, semi-continuous cultivation.

c NS24, natural sedimentation (about 24 h) to recycle cells for semi-continuous cultivation.

d CCa, semi-continuous cultivation with Ca^2+^ ions.

e CFe, semi-continuous cultivation with Fe^3+^ ions.

Compared to batch-fed cultivation, NS24 and CFe are significantly green and efficient; CFe, in particular, was at least 20% cheaper than batch-fed cultivation and SCC. When we changed the strains from G/Pel to G/Phy, the SCC, NS24, and CFe culture of G/Pel and G/Phy yielded similar results. This comprehensive evaluation indicates that NS24, CCa, and CFe consume fewer resources, decrease time cost, and produce less waste yeast than batch-fed cultivation and general SCC (Supplementary Tables S2, S3).

In sum, regardless of the fermentation process of G/Phy or G/Pel, when cells can be recycled, the same results are always obtained: less time cost, less resource consumption, and less waste yeast generation. In other words, the process of cell recycling is greener and more economical.

## Conclusion

We report a novel approach for P. pastoris GS115 protein production using a bioreactor, which reduces resource consumption, time cost, and waste yeast emission by recycling vigorous cells for repeated fermentation. Two proteins were tested using four processes to demonstrate the advantages of this approach. The approach has obvious advantages over batch-fed cultivation and general semi-continuous cultivation. Under the guidance of the principle of vitality cell recycling, the best process, CFe, only consumed 14.8.3 ± 0.0% glycerol, 97.8 ± 1.3% methanol, 55.0 ± 0.9 inorganic salts, and 81.5 ± 0.0% of time cost to produce the same amount of protein product as batch-fed cultivation, and waste yeast yield was only 77.0 ± 0.1% of that of batch-fed cultivation. Other processes designed under the principles of vitality cell recycling also consume significantly fewer resources, time cost, and less waste yeast emission, and the processes for protein production are more economical and greener than the classic *P*. *pastoris* culture process.

## Data Availability

The original contributions presented in the study are included in the article/Supplementary Material; further inquiries can be directed to the corresponding authors.
