# Supplementary material for: Green Process: Improved Semi-Continuous Fermentation of Pichia pastoris Based on the Principle of Vitality Cell Separation
Source: Front Bioeng Biotechnol. 2021 Nov 30;9:777774. doi: 10.3389/fbioe.2021.777774 (PMC8669635; doi:10.3389/fbioe.2021.777774)
Supplement: Supplementary file 1 [file DataSheet1.pdf]

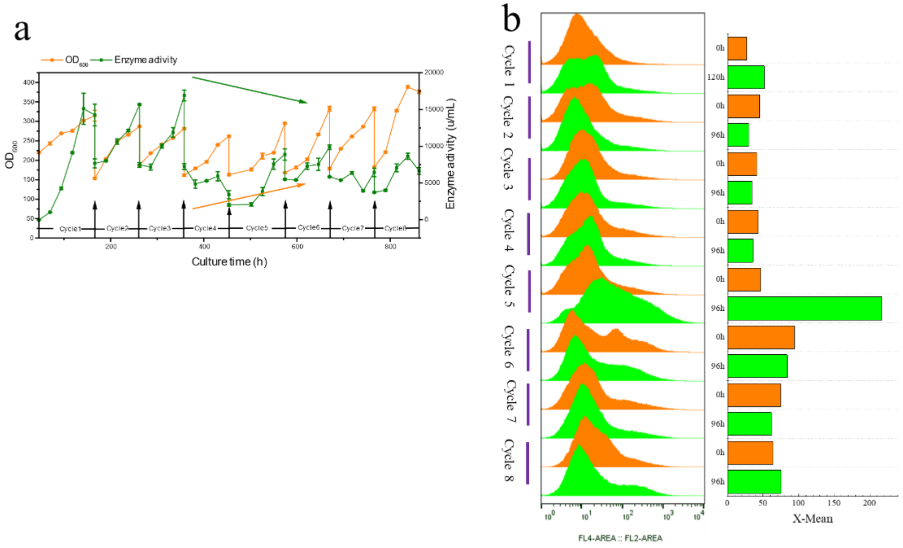


**Fig. S1** The semi-continuous cultivation of G/Phy. **a** Semi-continuous cultivation growth curve and enzyme activity curve of G/Phy. **b** G/Phy Cell vitality of semi-continuous cultivation.

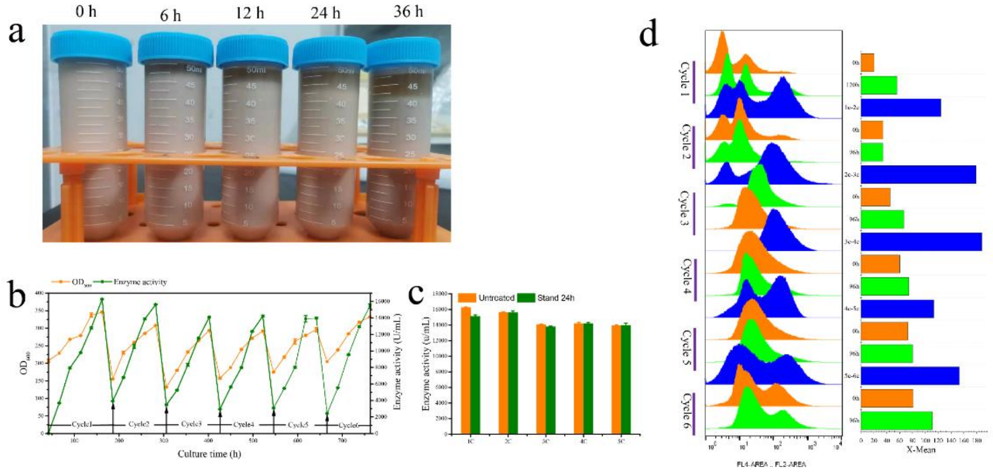


**Fig. S2** Natural sedimentation to recycle cells for semi-continuous cultivation. **a** The effect of natural sedimentation of G/Phy at different times. **b** Growth curve and enzyme activity curve of semi-continuous cultivation of G/Phy natural sedimentation 24h to recycle cells. **c** Comparison between untreated and after sedimentation enzyme activity of semi-continuous cultivation of G/Phy natural sedimentation 24h to recycle cells. **d** G/Phy cell vitality of natural sedimentation24h to recycle cells for semi-continuous cultivation. **Untreated:** End of the induction. **Stand 24 h:** After standing for 24 hours. The left is the histogram of flow cytometry, and the histogram migrates to the right, with increased fluorescence and decreased cell vitality. The right is the statistical data of fluorescence detected by flow cytometry at different time points.

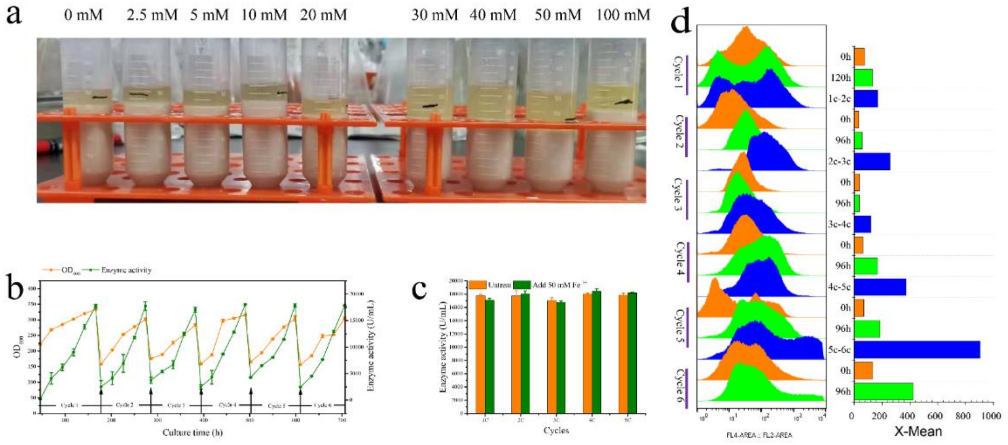


**Fig. S3** Adding $FeCl_3$ to accelerate cell sedimentation to recycle cells for semi-continuous cultivation. **a** The effect of sedimentation of G/Phy at different concentration $FeCl_3$. **b** Growth curve and enzyme activity curve of adding50mM $Fe^{3+}$ ions to accelerate G/Phy sedimentation to recycle cells. **c** Comparison between untreated and after sedimentation enzyme activity of adding 50mM $Fe^{3+}$ ions to accelerate G/Phy sedimentation to recycle cells.**d** G/Phy cell vitality of natural sedimentation24h to recycle cells for semi-continuous cultivation. **Untreated:** End of the induction. **Add 50 mM $Fe^{3+}$**: After adding 50mM $Fe^{3+}$ and standing for 12 hours. The left is the histogram of flow cytometry, and the histogram migrates to the right, with increased fluorescence and decreased cell vitality. The right is the statistical data of fluorescence detected by flow cytometry at different time points.

**Table S1.** Three processes assessment of G/pel. Detailed to each cycle.

| | Process | Cycle 1 | Cycle 2 | Cycle 3 | Cycle 4 | Cycle 5 | Cycle 6 | Cycle 7 | Cycle 8 | Average all cycle |
|---|---|---|---|---|---|---|---|---|---|---|
| product | SCC[a] | 65.4±2.9 | 69.9±0.1 | 68.1±0.3 | 66.9±0.1 | 70.2±0.8 | 68.7±0.3 | 62.4±0.1 | 100 | 71.5±11.8 |
| recovery (%) | NS24[b] | 82.3±0.7 | 84.6±2.2 | 92.5±0.0 | 83.4±0.2 | 85.1±0.0 | 92.0±0.1 | 85.0±0.2 | 100 | 88.1±6.1 |
| | CCa[c] | 88.0±0.1 | 82.0±0.2 | 85.7±0.1 | 82.3±0.2 | 100.0±0.2 | - | - | - | 87.6±7.4 |
| | CFe[d] | 85.5±0.1 | 84.8±0.2 | 90.9±0.0 | 83.2±0.3 | 85.4±0.0 | 82.9±0.1 | 84.8±0.1 | 100 | 87.2±7.3 |
| Cell recovery | SCC[a] | 37.6±0.5 | 37.9±0.2 | 41.4±0.3 | 29.5±0.3 | 42.6±0.2 | 35.5±0.3 | 34.0±0.4 | 0.0 | 32.3±13.7 |
| (%) | NS24[b] | 35.7±0.3 | 34.9±0.2 | 36.3±0.6 | 42.9±0.9 | 45.9±0.7 | 37.3±0.2 | 43.2±0.7 | 0.0 | 34.5±14.5 |
| | CCa[c] | 35.5±0.5 | 45.4±1.5 | 46.9±0.1 | 37.0±0.3 | 0.0 | - | - | - | 32.9±19.1 |
| | CFe[d] | 33.8±0.6 | 40.2±0.2 | 37.9±0.3 | 37.5±0.5 | 37.8±0.6 | 41.9±0.2 | 38.2±0.2 | 0.0 | 33.4±13.7 |
| Cell | SCC[a] | 348.33±4.07 | 263.33±1.53 | 256.00±2.00 | 320.33±2.89 | 272.00±1.00 | 351.83±3.21 | 368.8±3.90 | 343.33±5.05 | 315.5±45.0 |
| concentrtatio | NS24[b] | 164.33±3.55 | 179.00±5.57 | 153.33±1.53 | 163.67±1.53 | 141.00±1.00 | 160.33±9.22 | 167.5±1.80 | 298.00±3.77 | 178.4±49.6 |
| n of | CCa[c] | 184.67±4.48 | 142.33±4.25 | 161.67±2.47 | 135.67±1.61 | 242.17±0.29 | - | - | - | 173.3±42.9 |
| supernatant | CFe[d] | 187.33±2.02 | 176±1.73 | 173.33±3.21 | 168.00±3.49 | 169.17±3.40 | 143.83±0.29 | 158.33±4.48 | 298.5±1.00 | 184.3±47.9 |
| $\gamma_{x/m}$ (g/g) | SCC[a] | 0.180±0.005 | 0.227±0.003 | 0.231±0.004 | 0.286±0.004 | 0.238±0.002 | 0.315±0.007 | 0.326±0.006 | 0.294±0.007 | 0.262±0.051 |
| | NS24[b] | 0.178±0.006 | 0.295±0.005 | 0.211±0.007 | 0.250±0.01 | 0.233±0.008 | 0.276±0.005 | 0.233±0.008 | 0.234±0.013 | 0.239±0.036 |
| | CCa[c] | 0.181±0.007 | 0.161±0.013 | 0.189±0.002 | 0.178±0.011 | 0.205±0.002 | - | - | - | 0.183±0.016 |
| | CFe[d] | 0.172±0.001 | 0.236±0.006 | 0.246±0.002 | 0.251±0.009 | 0.254±0.007 | 0.235±0.006 | 0.245±0.004 | 0.249±0.004 | 0.236±0.027 |
| $\gamma_{p/m}$ (U/g) | SCC[a] | 262.65±22.21 | 275.37±1.66 | 249.00±3.29 | 125.50±1.65 | 165.44±9.83 | 180.57±3.14 | 183.65±1.71 | 178.29±3.61 | 202.56±53.20 |
| | NS24[b] | 274.44±11.40 | 280.70±11.66 | 258.81±45.7 | 306.94±4.34 | 261.43±2.29 | 269.73±675 | 325.95±4.08 | 271.88±2.37 | 281.24±23.37 |
| | CCa[c] | 278.59±2.15 | 312.38±5.38 | 289.78±4.38 | 270.19±375 | 271.50±2.34 | - | - | - | 284.49±17.42 |
| | CFe[d] | 278.39±2.09 | 285.81±5.41 | 290.03±3.08 | 294.63±4.99 | 290.98±2.86 | 271.99±3.13 | 293.85±3.24 | 278.52±1.84 | 285.53±8.33 |
| Time cost (h) | SCC[a] | 166 | 96 | 96 | 96 | 96 | 96 | 96 | 96 | 104.75 |
| | NS24[b] | 166 | 120 | 120 | 120 | 120 | 120 | 120 | 120 | 125.75 |
| | CCa[c] | 166 | 108 | 108 | 108 | 108 | - | - | - | 119.60 |

| | | | | | | | | | | |
|---|---|---|---|---|---|---|---|---|---|---|
| | CFe[d] | 166 | 108 | 108 | 108 | 108 | 108 | 108 | 108 | 115.25 |

[a]SCC, semi-continuous cultivation.

[b]NS24, natural sedimentation (about 24 h) to recycle cells for semi-continuous cultivation.

[c]CCa, semi-continuous cultivation with $Ca^{2+}$ ions.

[d]CFe, semi-continuous cultivation with $Fe^{3+}$ ions.

The fed-culture as 100% and other process compared with it.

Table S2. Three processes assessment of G/phy. Detailed to each cycle.

| | Process | Cycle 1 | Cycle 2 | Cycle 3 | Cycle 4 | Cycle 5 | Cycle 6 | Cycle 7 | Cycle 8 | Average all cycle |
|---|---|---|---|---|---|---|---|---|---|---|
| product recovery (%) | SCC[a] | 63.0±3.8 | 64.3±0.2 | 67.8±1.4 | 54.8±7.7 | 53.4±4.0 | 55.6±1.4 | 56.3±4.0 | 100.0 | 64.4±15.3 |
| | NS24h[b] | 83.5±0.0 | 83.4±0.1 | 84.3±0.0 | 83.7±0.1 | 86.9±0.1 | 100.00 | - | - | 90.0±6.2 |
| | CFe[c] | 90.3±0.1 | 84.2±0.7 | 89.1±0.3 | 82.2±0.1 | 89.7±0.2 | 100.00 | - | - | 89.2±6.5 |
| Cell recovery (%) | SCC[a] | 33.3±1.5 | 39.9±0.3 | 43.2±0.4 | 46.7±0.4 | 42.8±0.3 | 40.3±0.7 | 41.0±0.5 | 0.0 | 35.9±15.0 |
| | NS24h[b] | 30.7±0.1 | 36.0±0.0 | 40.3±0.0 | 45.0±0.4 | 41.8±1.0 | 0.0 | - | - | 32.3±16.6 |
| | CFe[c] | 32.3±0.0 | 39.6±0.4 | 41.9±0.5 | 39.3±0.1 | 38.7±0.1 | 0.0 | - | - | 32.0±16.0 |
| Supernatant ($OD_{600}$) | SCC[a] | 314.5±13.86 | 287.00±1.80 | 281.33±2.36 | 261.67±2.08 | 295.00±2.00 | 332.67±6.03 | 332.53±4.41 | 376.00±4.16 | 310.1±36.5 |
| | NS24h[b] | 234.67±0.29 | 170.67±3.01 | 208.33±1.89 | 213.67±4.25 | 185.83±3.79 | 332.67±0.58 | - | - | 224.3±57.6 |
| | CFe[c] | 226.50±2.31 | 231.33±3.75 | 232.33±1.15 | 267.33±2.60 | 161.33±1.15 | 305.50±3.46 | - | - | 237.4±48.0 |
| $\gamma_{x/methanol}$ (g/g) | SCC[a] | 0.163±0.015 | 0.193±0.004 | 0.174±0.004 | 0.158±0.004 | 0.194±0.004 | 0.232±0.008 | 0.223±0.007 | 0.269±0.007 | 0.201±0.038 |
| | NS24h[b] | 0.202±0.004 | 0.215±0.001 | 0.193±0.001 | 0.195±0.005 | 0.186±0.007 | 0.235±0.001 | - | - | 0.204±0.018 |
| | CFe[c] | 0.207±0.002 | 0.207±0.005 | 0.171±0.006 | 0.221±0.003 | 0.203±0.010 | 0.220±0.004 | - | - | 0.205±0.018 |
| $\gamma_{p/methanol}$ (U/g) | SCC[a] | 62764±6491 | 49602±2827 | 56540±4875 | < 0 | 36866±4377 | 28742±1815 | 10296±3161 | 19211±2520 | 44585±31279 |
| | NS24h[b] | 71463±205 | 63426±359 | 57439±332 | 60058±611 | 58171±647 | 68123±1511 | - | - | 53599±23835 |
| | CFe[c] | 77386±1429 | 79267±8325 | 71141±4382 | 80973±1922 | 73359±1610 | 80219±399 | - | - | 77057±3975 |

| | | | | | | | | | |
|---|---|---|---|---|---|---|---|---|---|
| Time cost (h) | SCC[a] | 166 | 96 | 96 | 96 | 96 | 96 | 96 | 96 | 104.75 |
| | NS24h[b] | 166 | 120 | 120 | 120 | 120 | 120 | - | - | 127.67 |
| | CFe[c] | 166 | 108 | 108 | 108 | 108 | 108 | - | - | 117.67 |

[a]SCC, semi-continuous cultivation.

[b] NS24, natural sedimentation (about 24 h) to recycle cells for semi-continuous cultivation.

[c]CFe, semi-continuous cultivation with $Fe^{3+}$ ions.

The fed-culture as 100% and other process compared with it.

Table S3. The assessment of G/Phy for three processes.

| | Glycerol consumption | Methanol consumption | inorganic salt consumption | Waste yeast | Time cost |
|---|---|---|---|---|---|
| Batch fed-cultivation[a] | 100 | 100 | 100 | 100 | 100 |
| SCC[b] | 26.8±0.3 | 177.1±2.4 | 120.7±1.6 | 138.5.4±5.3 | 134.2±5.3 |
| NS24h[c] | 20.8±0.2 | 104.3±0.8 | 54.9±0.5 | 74.9.5±0.7 | 95.3±0.7 |
| CFe[d] | 18.6±0.2 | 93.5±1.2 | 61.7±0.8 | 70.8±0.1 | 78.8±0.7 |

[a]The consumption and waste yeast emissions, Batch fed-culture as 100% and other process compared to it.

[b]SCC, semi-continuous cultivation.

[c] NS24, natural sedimentation (about 24 h) to recycle cells for semi-continuous cultivation.

[d]CFe, semi-continuous cultivation with $Fe^{3+}$ ions.
